# An all-natural bioinspired structural material for plastic replacement

**DOI:** 10.1038/s41467-020-19174-1

**Published:** 2020-11-03

**Authors:** Qing-Fang Guan, Huai-Bin Yang, Zi-Meng Han, Zhang-Chi Ling, Shu-Hong Yu

**Affiliations:** Division of Nanomaterials & Chemistry, Hefei National Laboratory for Physical Sciences at the Microscale, Institute of Energy, Hefei Comprehensive National Science Center, CAS Center for Excellence in Nanoscience, Department of Chemistry, Institute of Biomimetic Materials & Chemistry, University of Science and Technology of China, Hefei, 230026 China

**Keywords:** Polymers, Bioinspired materials, Mechanical properties

## Abstract

Petroleum-based plastics are useful but they pose a great threat to the environment and human health. It is highly desirable yet challenging to develop sustainable structural materials with excellent mechanical and thermal properties for plastic replacement. Here, inspired by nacre’s multiscale architecture, we report a simple and efficient so called “directional deforming assembly” method to manufacture high-performance structural materials with a unique combination of high strength (281 MPa), high toughness (11.5 MPa m^1/2^), high stiffness (20 GPa), low coefficient of thermal expansion (7 × 10^−6^ K^−1^) and good thermal stability. Based on all-natural raw materials (cellulose nanofiber and mica microplatelet), the bioinspired structural material possesses better mechanical and thermal properties than petroleum-based plastics, making it a high-performance and eco-friendly alternative structural material to substitute plastics.

## Abstract (web summary)

It is desirable yet challenging to develop sustainable structural materials to replace petroleum-based plastics. Here, the authors report a facile assembly method for manufacturing high-performance structural materials with a unique combination of high strength, toughness and stiffness.

## Introduction

Modern life relies closely on plastics, yet the vast majority of these plastics are derived from petrochemicals^1–3^. Thus, there are environmental concerns associated with both the raw materials used to make them and their end­-of­-life options^4–8^. Although there is no panacea for these complex environmental problems^1^, one option is to develop sustainable high-performance structural materials to partly substitute petroleum-based plastics. However, until now, sustainable structural materials constructed from bio-resources suffer from either limited mechanical properties or complex manufacturing processes, resulting in high cost and difficulty to produce in large scale^1,2,9^. Therefore, it is of great importance to introduce an advanced strategy to design and manufacture sustainable high-performance structural materials^9–11^.

Biomimetic design has been a promising strategy to improve the properties of structural materials^12,13^. Due to the hierarchically ordered structure at multiscale levels, many successful cases of improving mechanical properties have been achieved through this strategy^13–17^. One of the most effective models is the nacre with a “brick-and-mortar” microstructure, which combines mutually exclusive properties (strength and toughness) in one material with all-natural raw materials under moderate conditions^18–22^. From the perspective of plastic replacement, through biomimetic design, the sustainable structural materials with the brick-and-mortar structure can exceed the limitation of poor mechanical properties. Moreover, if complex manufacturing processes can be simplified, all-natural bioinspired high-performance structural materials will be the good substitutes for petroleum-based plastics.

Here, we develop a simple and robust method with biomimetic design, named as “directional deforming assembly”, which can be applied for further scale-up in an efficient way. By this directional deforming assembly method, we successfully manufacture all-natural bioinspired high-performance structural materials from prepared cellulose nanofiber (CNF) and TiO_2_-coated mica microplatelet (TiO_2_-mica) composite hydrogel. The obtained structural material has better mechanical and thermal properties than those of petroleum-based plastics, making it a strong competitor to petroleum-based plastics. Mass production, good processability, and tunable coloration allow it to be used to fabricate a series of advanced, beautiful, and durable structural materials to replace plastics, for example, structural support for high-end personal electronic devices.

## Results

### Material design and fabrication strategy

Inspired by brick-and-mortar microstructure of nacre, we employ hierarchically ordered structure design at multiscale levels based on all-natural material^18^. As one of the most abundant all-green resources on Earth, CNF, a high-performance one-dimensional (1D) nanoscale building block, can be derived from plants or produced by bacteria (Supplementary Fig. 1)^23,24^. It possesses high strength (at least 2 GPa), a low coefficient of thermal expansion (CTE) (1 × 10^−7^ K^−1^), and abundant hydroxyl and carboxyl groups on the surface, indicating it to be an ideal biopolymer matrix for brick-and-mortar structure^24–27^. Mica microplatelet, exfoliated from natural mica, is an all-natural two-dimensional (2D) inorganic building block^28^. Based on mica microplatelet, TiO_2_-coated mica microplatelet (TiO_2_-mica) is commercially available, which has been widely used in pigments or cosmetics because of its special beautiful pearlescent color. Besides, TiO_2_-mica consists of TiO_2_ nanograins with diameters ranging from 10 to 100 nm at the surface of mica microplatelet, in accordance with those of aragonite platelets in nacre (Supplementary Fig. 2)^18^. Therefore, TiO_2_-mica is a proper 2D inorganic building block to fabricate nacre-mimetic sustainable structural materials.

In order to achieve the highly ordered brick-and-mortar structure, we designed the process of dramatically reducing the thickness of the hydrogel while the size on in-plane directions keep unchanged. By directly pressing hydrogel of 2D and 1D building blocks, the 2D building blocks (bricks) can achieve highly uniform orientations and 1D nanoscale building blocks (mortars) can evenly distribute between the bricks. This method, named as “directional deforming assembly” method, is a robust and simple one-step method to assemble the highly ordered brick-and-mortar structure directly, which can make the preparation of large-sized nacre-mimetic bulk much faster and low cost. This simple and robust directional deforming assembly method can be used to directly construct high-performance sustainable structural materials with the highly ordered brick-and-mortar structure.

**Fig. 1:**
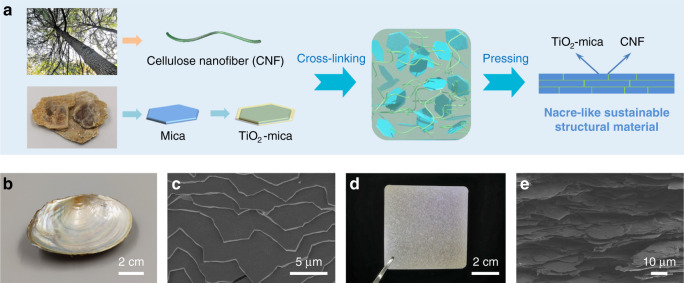
Fabrication and characterization of all-natural bioinspired structural material. **a** Schematic illustration of directional deforming assembly method to manufacture all-natural bioinspired high-performance structural material. **b** *Anodonta woodiana*. **c** Fracture surface of *Anodonta woodiana*. **d** All-natural bioinspired structural material. **e** Fracture surface of all-natural bioinspired structural material.

Figure 1a shows a schematic of our efficient directional deforming assembly method to fabricate all-natural bioinspired structural materials. The 2D TiO_2_-mica is pretreated by (3-aminopropyl)triethoxysilane (APTES), which can facilitate the interfacial interaction between the 2D TiO_2_-mica and the 1D CNF. CNF is fully mixed with APTES-treated TiO_2_-mica and crosslinked by Ca^2+^ to form hydrogel, which can be directly pressed into the structural material by directional deforming assembly. During the directional deforming assembly, directional orientations of 2D TiO_2_-mica and uniform distribution of 1D CNF between the TiO_2_-mica can be achieved, forming a highly ordered brick-and-mortar structure. Through the above facile process from a molecular to a macroscopic level, all-natural bioinspired structural materials with a low density of ~1.7 g cm^−3^ are obtained, sharing similarities with natural nacre in appearance, brick-and-mortar structure, and nanograins at the surface of platelets (Fig. 1b–e and Supplementary Fig. 3).

### Mechanical performances and toughening mechanisms analysis

**Fig. 2:**
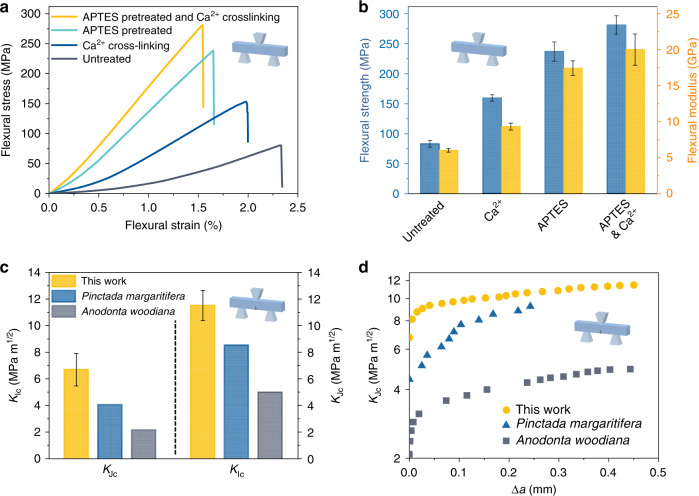
Mechanical properties of the all-natural bioinspired structural material. **a** Flexural stress–strain curves of all-natural bioinspired structural material with different pretreatments. **b** Comparison of flexural strength and stiffness of all-natural bioinspired structural material with different pretreatments. Error bars represent standard deviation. **c** Fracture toughness for crack initiation (*K*_Ic_) and steady-state (*K*_Jc_) of the all-natural bioinspired structural material and some natural nacre^18^. Error bars represent standard deviation. **d** Rising crack-extension resistance curves (evaluated by the steady-state fracture toughness *K*_Jc_) of the all-natural bioinspired structural material and some natural nacre^18^.

To demonstrate the validity of surface chemical modification in the brick-and-mortar structure, the mechanical properties of the all-natural bioinspired structural materials are then systematically studied and compared to those of the composites without APTES pretreatment or Ca^2+^ crosslinking. As shown in Fig. 2a, b, the flexural strength and flexural modulus of untreated composite can only reach ~83 and ~6 GPa, respectively, because only hydrogen bonds exist in untreated composite and the interfacial interaction between CNF and TiO_2_-mica is poor. After being pretreated by APTES, hydroxyl groups on the TiO_2_ nanograins surface react with silane groups of APTES to form a silanized surface, which can facilitate the attachment of TiO_2_-mica to CNF^29^. At the same time, carboxyl groups of CNFs are crosslinked by the Ca^2+^, which forms a powerful ionic bond network and further enhances the interaction between CNFs (Supplementary Fig. 4). Furthermore, through directional deforming assembly, the orientation and orderly arrangement of APTES-treated TiO_2_-mica in the hydrogel are achieved, forming the robust highly ordered brick-and-mortar structure. Thus, after surface chemical modification and directional deforming assembly, the all-natural bioinspired structural material demonstrates a high flexural strength of 281 MPa and a high flexural modulus of 20 GPa, which are more than three times higher than those of untreated composite (Fig. 2a, b).

A long-standing challenge in engineering material design is the conflict between strength and toughness, which are in general mutually exclusive in most artificial materials^17,18,30^. However, through the design of a highly ordered brick-and-mortar structure with surface chemical modification and toughening mechanisms at multiscale levels, the all-natural bioinspired structural material reconciles its strength and toughness. As shown in Fig. 2c, d, the fracture toughness, *K*_Ic_, describes the resistance to a crack initiation and is measured to be ~6.7 MPa m^1/2^, much higher than those of natural *Anodonta woodiana* nacre (~2.1 MPa m^1/2^) and *Pinctada margaritifera* nacre (~4.0 MPa m^1/2^). Its good fracture toughness illustrates that our designed highly ordered brick-and-mortar structure can resist crack initiation effectively. Although *K*_Ic_ can be an evaluation of the resistance to a crack initiation, it is unable to evaluate the multiple extrinsic toughening mechanisms in the stage of crack propagation, which greatly contribute to the dissipation of energy^21,22^. According to the *J*–*R* curve approach, the *R*-curve behavior can represent fracture toughness actually increases with crack extension and the multiple extrinsic toughening mechanisms, and steady-state fracture toughness, *K*_Jc_, can describe the maximum fracture toughness of materials. Thus, we use the *J*–*R* curve approach to fully evaluate toughness. Figure 2c, d shows that the all-natural bioinspired structural material demonstrates a *K*_Jc_ of 11.5 MPa m^1/2^, which is significantly higher than those of natural *A. woodiana* nacre (~5.0 MPa m^1/2^) and natural *P. margaritifera* nacre (~8.5 MPa m^1/2^). These results strongly confirm that the all-natural bioinspired structural material possesses both high strength and toughness, which can be ascribed to our multiscale design of the highly ordered brick-and-mortar structure with surface chemical modification by the directional deforming assembly method.

**Fig. 3:**
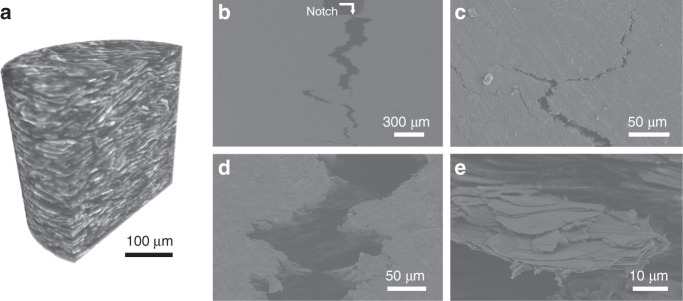
Brick-and-mortar structure and multiscale extrinsic toughening mechanisms. **a** 3D reconstruction of the all-natural bioinspired structural material derived from X-ray microtomography. **b** Fracture surface of an all-natural bioinspired structural material sample that shows long-range crack deflection. **c** Crack deflection, branching, bridging, and multiple cracking towards the end of the crack path. **d** Pulling out of TiO_2_-mica microplatelets. **e** Details of the fracture surface showing TiO_2_-mica microplatelet’s pulling out, delamination, CNF bridging, and stretching.

In order to further analyze the multiscale extrinsic toughening mechanisms, we carried out the three-dimensional (3D) reconstruction of brick-and-mortar structure and observation of single-edge notched bending specimens. Similar to natural nacre, the all-natural bioinspired structural material has a robust highly ordered brick-and-mortar structure, where CNF and TiO_2_-mica bond together tightly (Fig. 3a and Supplementary Movie 1). As shown in Fig. 3b, the crack that initiates from the notch propagates along a tortuous path in the all-natural bioinspired structural material. Besides this typical crack deflection, the laminated nacre-mimetic brick-and-mortar structure also leads to delamination, crack branching, multiple cracking, and crack bringing at the crack tip, which effectively relieve the locally high stress (Fig. 3c–e). Moreover, the TiO_2_ nanograins in the surface of TiO_2_-mica lead to efficient energy dissipation by frictional sliding during TiO_2_-mica pull-out (Supplementary Fig. 3). In addition, the platelet–biopolymer interface strength is greatly improved by surface chemical modification, which also plays a key role in stress transfer and damage tolerance. All the proposed multiscale extrinsic toughening mechanisms derived from the hierarchical brick-and-mortar structure contribute to the load redistribution and toughness enhancement in the all-natural bioinspired structural material.

### Comparison of thermal properties

**Fig. 4:**
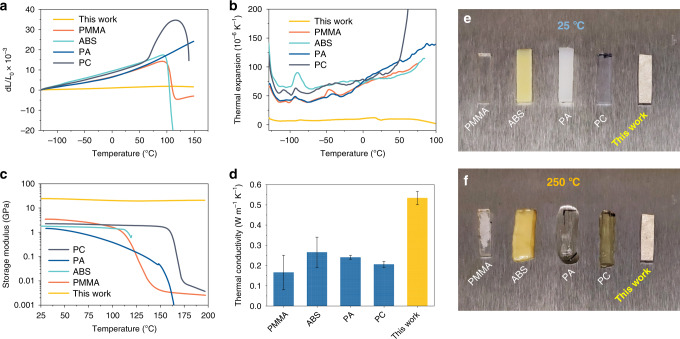
Comparison of thermal properties of all-natural bioinspired structural material with widely used plastics. **a** Comparison of thermal expansion of the all-natural bioinspired structural material with widely used petroleum-based plastics. **b** Comparison of coefficient of thermal expansion (CTE) of the all-natural bioinspired structural material with widely used petroleum-based plastics. **c** Comparison of storage modulus of the all-natural bioinspired structural material with widely used petroleum-based plastics. **d** Comparison of thermal conductivity of the all-natural bioinspired structural material with widely used petroleum-based plastics^31^. Error bars represent standard deviation. **e**, **f** Thermal stability experiment. Comparison of the all-natural bioinspired structural material with widely used petroleum-based plastics at **e** 25 °C and **f** 250 °C. Compared to 25 °C, petroleum-based plastics have already fully softened at 250 °C, while all-natural bioinspired structural material still shows no visible change. PMMA polymethyl methacrylate, ABS acrylonitrile butadiene styrene, PA polyamide, PC polycarbonate.

The thermal behavior of structural materials is critical to a variety of applications, especially for high or variable temperatures in service conditions. Due to the bad thermal behavior (e.g., poor thermal stability and softening at high temperature), the application of plastics is limited^31^. However, the all-natural bioinspired structural material can exceed the limitation of bad thermal behavior, which can be attributed to the high crystallinity of CNF, good thermal stability of TiO_2_-mica, and the highly ordered brick-and-mortar structure with surface chemical modification, making it a high-performance alternative for plastic replacement. As shown in Fig. 4a, the dimensions of plastics change greatly as the temperature increases, while our structural material is almost unchanged under temperatures ranging from −130 to 150 °C. Therefore, the all-natural structural material demonstrates excellent thermal dimensional stability and its CTE is ~7 × 10^−6^ K^−1^ at room temperature (25 °C), more than 10 times lower than those of plastics (Fig. 4b). In practical application, large CTE will result in thermal stresses that often cause structure failure, so low CTE is an important safeguard for long-term use of the structural material at variable temperatures in service conditions. Due to good thermal stability and low CTE, the mechanical properties of all-natural structural material remain nearly unchanged at variable temperatures. Its storage modulus can keep ~20 GPa and remains almost steady as temperatures ranging from 25 to 200 °C, which are higher and more stable than those of plastics (Fig. 4c and Supplementary Fig. 5). More intuitively, it can be observed that all typical plastics have already fully softened at 250 °C, while the all-natural structural material still shows no visible change compared to 25 °C (Fig. 4e, f). Moreover, its thermal diffusion is also higher than those of plastics, which is conducive to heat dissipation to further ensure reliability for practical application (Fig. 4d and Supplementary Figs. 6 and 7)^31^. All thermal properties mentioned above illustrate that as a kind of emerging structural material, our all-natural bioinspired structural material is safer and more reliable than plastics, making it a sustainable, lightweight, high-performance alternative at high or variable temperatures to substitute plastics.

### Performance advantages for plastic replacement

**Fig. 5:**
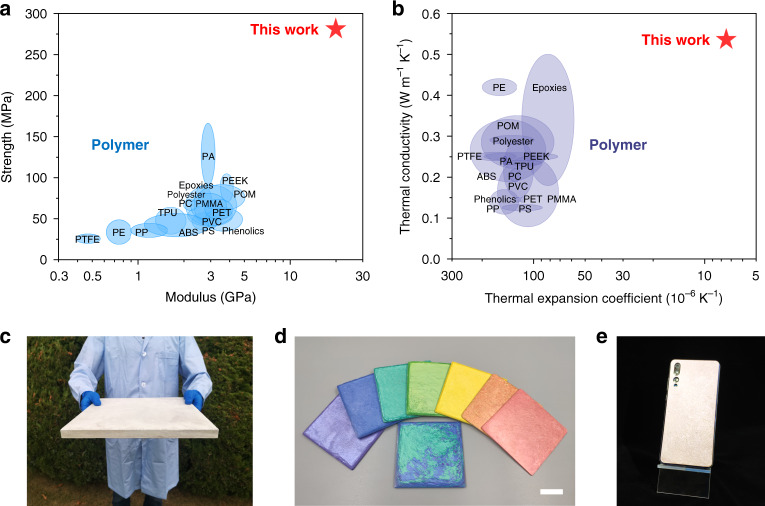
Comparison of mechanical and thermal properties of all-natural bioinspired structural material with typical polymers. **a** Ashby diagram of ultimate strength vs. modulus for all-natural bioinspired structural material compared with typical polymers^31^. **b** Ashby diagram of thermal conductivity vs. CTE for all-natural bioinspired structural material compared with typical polymers^31^. **c** Large-sized all-natural bioinspired structural material with a volume of 310 × 300 × 18 mm^3^. **d** All-natural bioinspired structural materials with different colors. Scale bar, 2 cm. **e** Mobile phone shell produced from the all-natural bioinspired structural material. PTFE polytetrafluoroethylene, PE polyethylene, PP polypropylene, TPU thermoplastic urethane, PVC polyvinyl chloride, PS polystyrene, PET polyethylene terephthalate, PEEK poly(ether ether ketone), POM polyformaldehyde.

As a kind of environmentally friendly structural material, the all-natural bioinspired structural material has a unique combination of excellent mechanical and thermal properties (Fig. 5a, b and Supplementary Fig. 8). As shown in Fig. 5a, both of its strength and modulus are much higher than those of polymers, which are twice and five times higher than those of most plastics, respectively. Meanwhile, it is much harder and tougher than typical plastics, indicating it is durable and not easily deformed for advanced applications (Supplementary Fig. 9 and Supplementary Table 1). Thus, our all-natural bioinspired structural material is lightweight, strong, tough, and hard, providing adequate assurance of mechanical performance as structural material for plastic replacement. As shown in Fig. 5b, both of its CTE and thermal diffusion are much better than those of polymers. As a high-performance structural material with low CTE, great thermal stability, no softening at high temperature, and good thermal diffusion, the all-natural bioinspired structural material is very safe and reliable at high or variable temperatures to substitute plastics. Moreover, because our directional deforming assembly method is effective and scalable, mass production of all-natural bioinspired structural material can be achieved (Fig. 5c). Basing on different commercially available raw materials (e.g., TiO_2_-mica, Fe_2_O_3_-mica), we can fabricate a variety of all-natural bioinspired structural materials with different colors (Fig. 5d). As shown in Fig. 5e, the obtained structural material has good processability, which can be fabricated into desired shape and size, showing a vast potential to replace plastics for practical applications, for example, structural support for high-end personal electronic device.

## Discussion

In summary, our biomimetic design of the highly ordered brick-and-mortar structure provides key ideas to fabricate sustainable structural materials for plastic replacement by the directional deforming assembly method. Through this simple and efficient directional deforming assembly of pre-prepared CNF and TiO_2_-mica hydrogel with biomimetic design, we can manufacture an all-natural bioinspired structural material, which has better mechanical and thermal properties than petroleum-based plastics. As a sustainable, high-performance structural material, mass production of bulk all-natural bioinspired structural material can be fabricated by this method, making it a strong competitor to plastics. Moreover, this directional deforming assembly method opens up the possibility to fabricate sustainable bioinspired structural materials in bulk and practical form by expanding it to other 1D and 2D building blocks. We anticipate that this kind of all-natural bioinspired structural materials with excellent mechanical and thermal properties can play a key role in plastic replacement.

## Methods

### Fabrication of all-natural bioinspired structural materials

All reagents and raw materials were commercially available. In a typical fabrication, 15 g of TiO_2_-coated mica microplatelet (TiO_2_-mica; Fujian Kuncai Material Technology, KC100W) was dispersed in 200 mL of deionized water (DIW), added 10 mL of (3-aminopropyl)triethoxysilane (APTES), and stirred at room temperature for 1 day as pretreatment. After pretreatment, the TiO_2_-mica dispersion was filtered and washed three times with DIW. APTES-treated TiO_2_-mica and 500 mL of TEMPO-oxidized^32^ CNF (Micro-Nano New Materials Technology Co. Ltd, 3 wt%) were mixed and stirred at room temperature for 10 min. The mixed hydrogel was then crosslinked by spraying CaCl_2_ (150 mL, 0.5 mol L^−1^). At last, the crosslinked hydrogel was pressed with a pressure of 1 MPa for about 12 h, and then a pressure of 100 MPa was applied at 80 °C for about 1 h to obtain all-natural bioinspired structural material.

### Characterization

Scanning electron microscopy images were taken with a Carl Zeiss Supra 40 field emission scanning electron microscope at an acceleration of 5 kV. Transmission electron microscopy images were acquired using a Hitachi HT7700 transmission electron microscope. Fourier transform infrared spectroscopy (FT-IR) spectra were acquired by a Bruker Vector-22 FT-IR spectrometer at room temperature. X-ray diffraction of patterns were carried out on a PANalytical X’pert PRO MRD X-ray diffractometer equipped with Cu *K*α radiation (*λ* = 1.54056 Å). The X-ray microtomography was conducted on Zeiss Xradia 520 for the 3D microstructural information. The raw data were reconstructed using the software Dragonfly by assembling the static images in sequence. Atomic force microscope images were taken with a Bruker Dimension FastScan.

### Mechanical performance testing

Three-point bending tests and single-edge notched bend (SENB) tests were carried out on an Instron 5565A universal testing machine, which was carried out at room temperature with a support span of 12.5 mm. For three-point bending tests, the samples were carefully cut with the size of about 30 mm ×  2 mm × 2 mm and the test was performed at a loading rate of 1.0 mm min^−1^. For SENB tests, the samples with the size of about 30 mm × 2 mm × 2 mm were notched to approximately half of their widths using a diamond saw (~300 μm), and then the notch was sharpened by slightly sliding a razor blade repeatedly. The loading rate was 1 μm s^−1^ for SENB samples. Hardness of samples was measured by Shore D Durometer. Each kind of material was tested at least five times. For all the mechanical tests, the applied loading direction was perpendicular to platelets of TiO_2_-mica.

### Thermal performance testing

CTEs of bioinspired structural materials and plastics were measured by NETZSCH TMA 402F3 and the tests were carried out from −130 to 150 °C. For CTE, the direction of bioinspired structural materials was parallel to platelets of TiO_2_-mica.

Dynamic mechanical analysis (DMA) measurements were performed on a DMA Q800 instrument using a three-point bending. The samples of bioinspired structural materials and plastics were about 3 mm thick, 10 mm wide, and 20 mm long. A pre-load of 0.05 N was applied before the measurement and the tests were carried out from 30 to 190 °C. The applied loading direction of bioinspired structural materials was perpendicular to platelets of TiO_2_-mica. The storage modulus and loss factor (tan *δ*) were measured.

Thermal conductivity and thermal diffusivity of bioinspired structural materials were measured by HotDisk 2500s at 25 °C for direction which was perpendicular to platelets of TiO_2_-mica.

### Calculation

Density (*ρ*) of bioinspired structural materials was calculated by first treating the material into a cuboid, and then using the equation *ρ* = mass/volume.

CTE was calculated by the equation CTE = Δ*L*/(*L* Δ*T*) K^−1^, where *L* represents the original length of sample, Δ*L* the change in length, and Δ*T* the change of temperature.

The fracture toughness was calculated by SENB test. Fracture toughness for crack initiation (*K*_Ic_) was calculated by

1 $$K_{{\mathrm{Ic}}} = \frac{{P_{{\mathrm{Ic}}}S}}{{BW^{3/2}}}f\left( {a/W} \right),$$

where *P*_Ic_ is the maximum load in SENB test, *S* the span, *B* the thickness of the SENB specimen, *W* the width, and *a* the length of the pre-crack. The function *f*(*a*/*W*) is given by

2 $$f(a/W) = \frac{{3(a/W)^{1/2}\left[ {1.99 - \left( {a/W} \right)\left( {1 - a/W} \right)\left( {2.15 - 3.93a/W + 2.7(a/W)^2} \right)} \right]}}{{2(1 + 2a/W)(1 - a/W)^{3/2}}}.$$

The maximum fracture toughness (*K*_Jc_) is determined by

3 $$K_{{\mathrm{Jc}}} = [(J_{{\mathrm{el}}} + J_{{\mathrm{pl}}})E^{\prime}]^{1/2},$$

where *J*_el_ represents the elastic component of *J*-integral, *J*_pl_ the plastic component of *J*-integral and *E*′ is given by

4 $$E^{\prime} = E(1 - v^2),$$

where *E* represents the elastic modulus and *ν* the Poisson’s ratio.

The elastic component *J*_el_ was based on linear elastic fracture mechanics.

5 $$J_{{\mathrm{el}}} = \frac{{K_{{\mathrm{Ic}}}^2}}{{E^{\prime}}}.$$

The plastic component *J*_pl_ was calculated by

6 $$J_{{\mathrm{pl}}} = \frac{{2A_{{\mathrm{pl}}}}}{{B(W - a)}}.$$

## Supplementary information

Supplementary Information

Description of Additional Supplementary Files

Supplementary Movie 1

## Data Availability

The data that support the findings of this study are available from the corresponding author upon reasonable request.
