# Supplementary material for: An all-natural bioinspired structural material for plastic replacement
Source: Nat Commun. 2020 Nov 3;11:5401. doi: 10.1038/s41467-020-19174-1 (PMC7642342; doi:10.1038/s41467-020-19174-1)
Supplement: Supplementary file 1 — Supplementary Information [file 41467_2020_19174_MOESM1_ESM.pdf]

Supplementary Information for

# An all-natural bioinspired structural material for plastic replacement

Qing-Fang Guan[†], Huai-Bin Yang[†], Zi-Meng Han[†], Zhang-Chi Ling, Shu-Hong Yu*

* Correspondence to: Shu-Hong Yu (shyu@ustc.edu.cn).

† These authors contributed equally to this work.

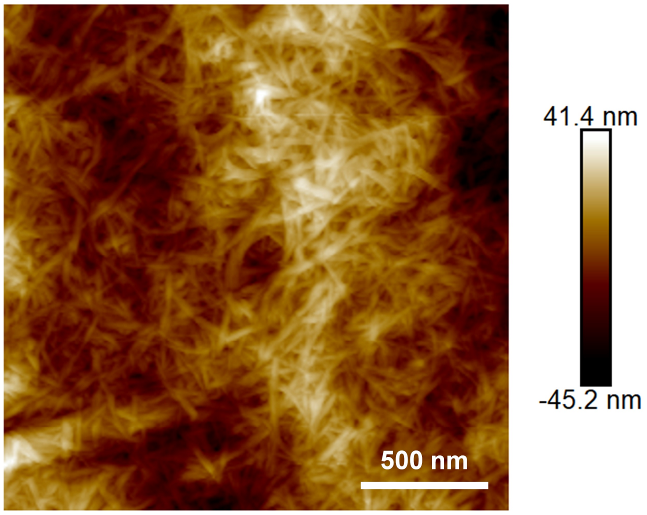


**Supplementary Figure 1 | Atomic force microscope (AFM) image of TEMPO-oxidized cellulose nanofiber (CNF).**

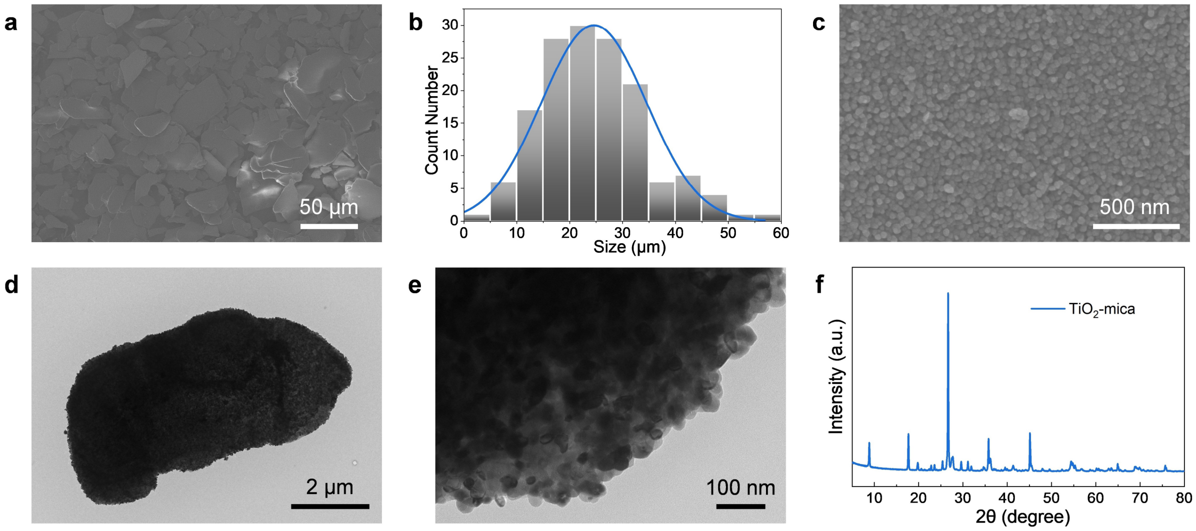


**Supplementary Figure 2 | Characterization of $TiO_2$ coated mica microplatelet ($TiO_2$-mica).** **a**, Scanning electron microscope (SEM) image of micro-sized $TiO_2$-mica platelets. The average lateral size of $TiO_2$-mica is about 25 μm, and the thickness is about 1 μm. **b**, The statistical lateral size of the $TiO_2$-mica microplatelet, showing the lateral size ranging from 4 μm to 60 μm. **c**, SEM image of the surface of $TiO_2$-mica microplatelet, showing the $TiO_2$-mica consisted of $TiO_2$ nanograins with diameters ranging from 10 to 100 nm on its surface. **d**, Transmission electron microscope (TEM) image of the micro-sized $TiO_2$-mica platelet. **e**, TEM image of the surface of $TiO_2$-mica microplatele, showing the $TiO_2$ nanograins were evenly coated on the surface of $TiO_2$-mica. **f**, XRD pattern of $TiO_2$-mica microplatelets.

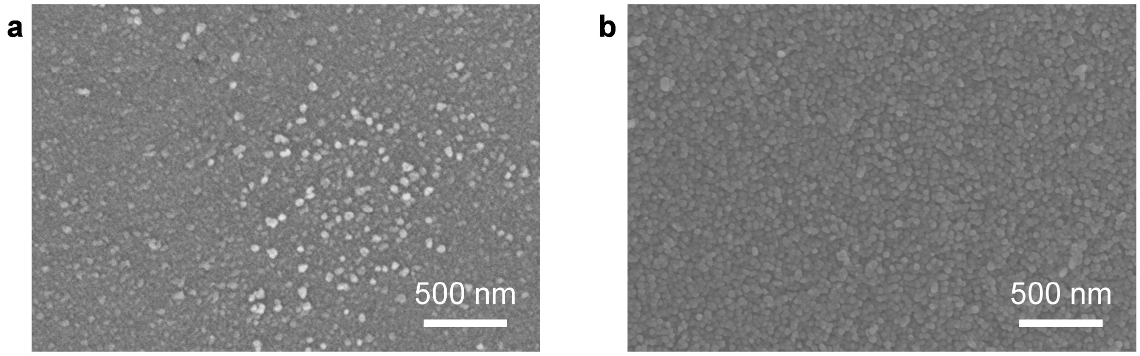


**Supplementary Figure 3 | The comparison of the unique connected-nanograin structure between natural nacre and the all-natural bioinspired structural material.** **a**, Surface of *Anodonta woodiana*. **b**, Surface of the all-natural bioinspired structural material.

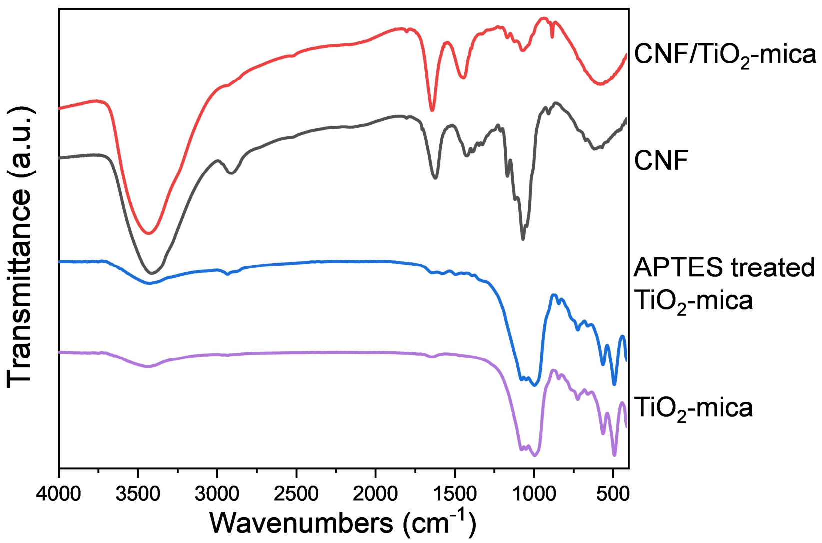


**Supplementary Figure 4 | Comparison of Fourier transform infrared spectroscopy (FT-IR).**

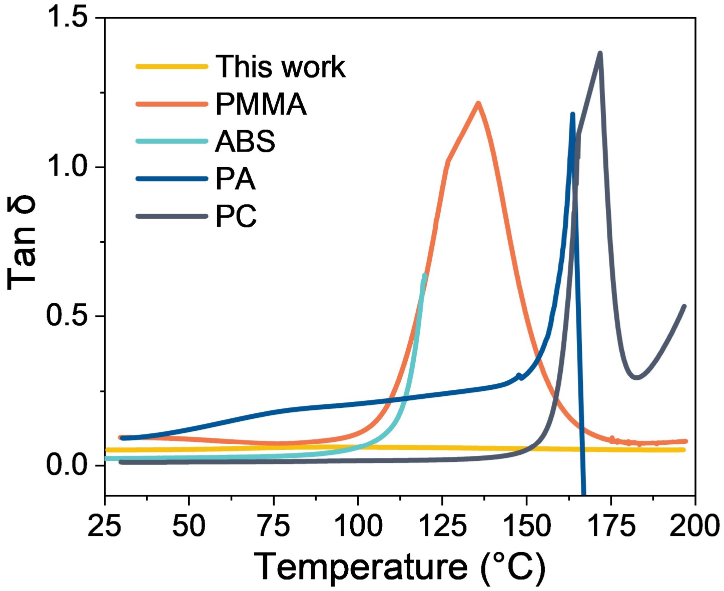


**Supplementary Figure 5 | Comparison of loss factor (tan δ).** The curves show that Tan δ of the all-natural bioinspired structural material is more stable than widely used petroleum-based plastics under temperature ranging from 30 ℃ to 190 ℃.

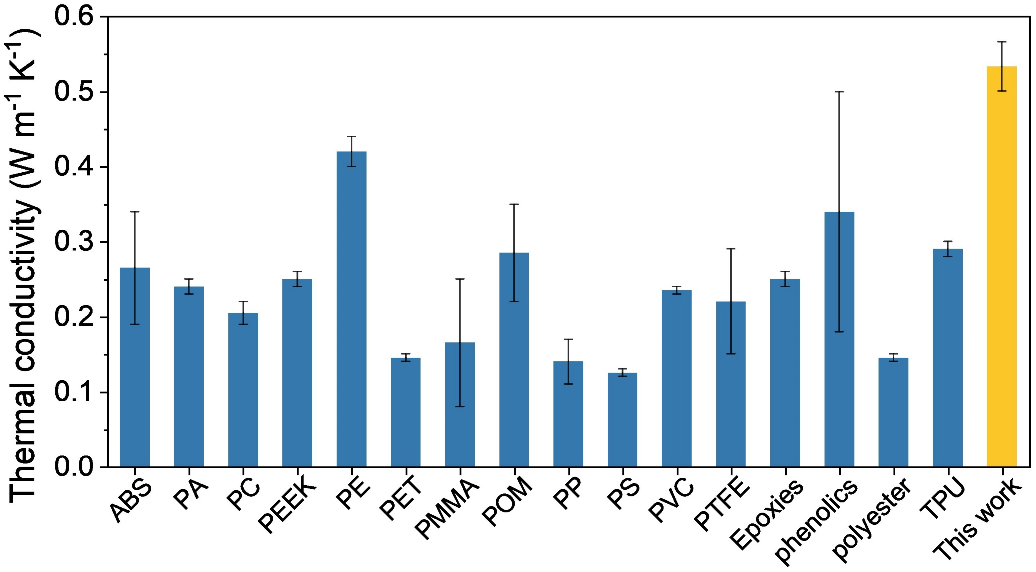


**Supplementary Figure 6 | Comparison of thermal conductivity.** Comparison of thermal conductivity of the all-natural bioinspired structural material with typical polymers[1].

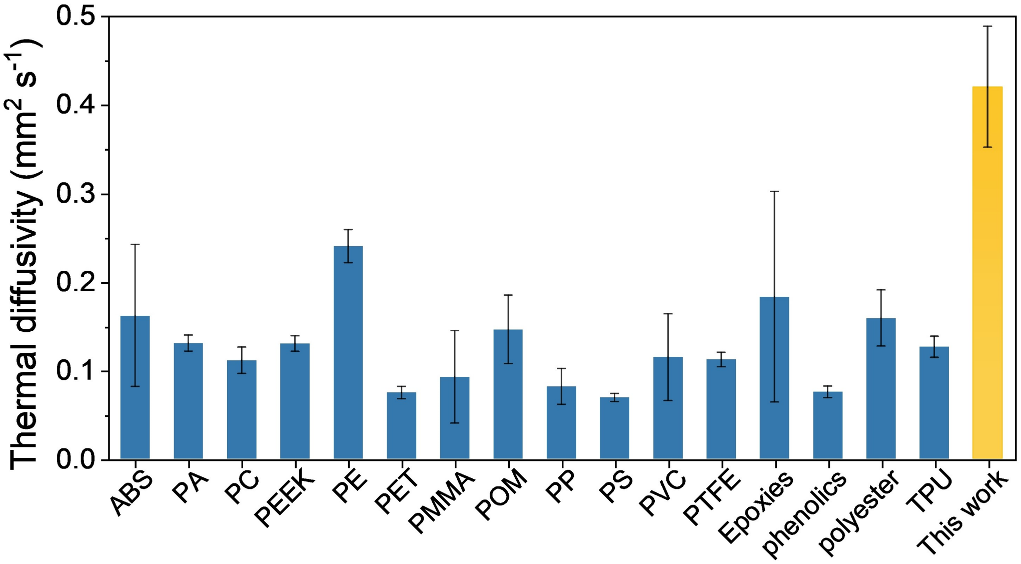


**Supplementary Figure 7│Comparison of thermal diffusivity.** Comparison of thermal diffusivity of the all-natural bioinspired structural material with typical polymers[1].

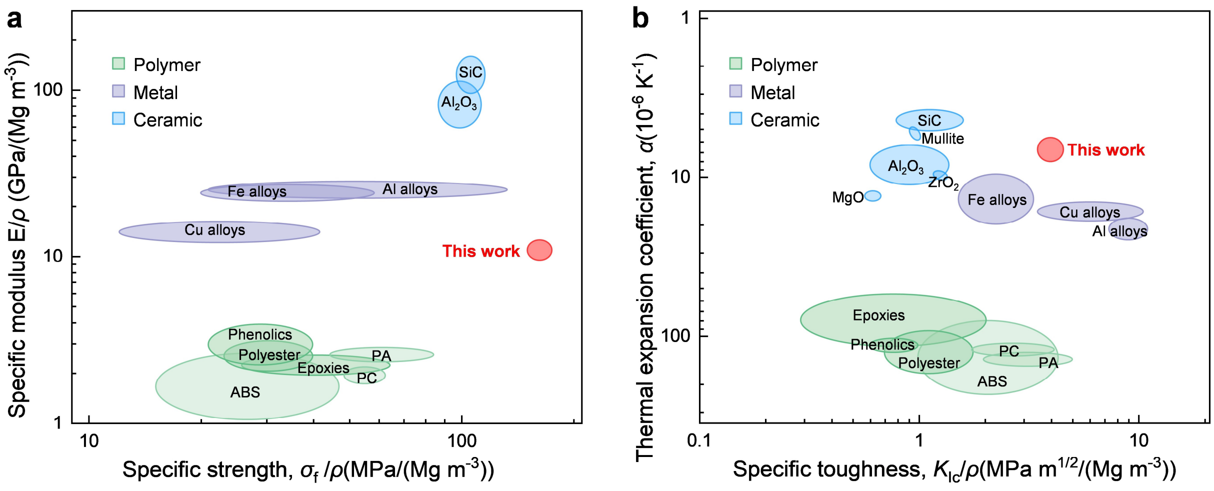


**Supplementary Figure 8 | Comparison of thermal and mechanical properties of all-natural bioinspired structural material with typical polymers, metals, and ceramics.** a, Ashby diagram of specific modulus versus specific yield strength[1,2]. b, Ashby diagram of thermal expansion versus specific fracture toughness[1,2].

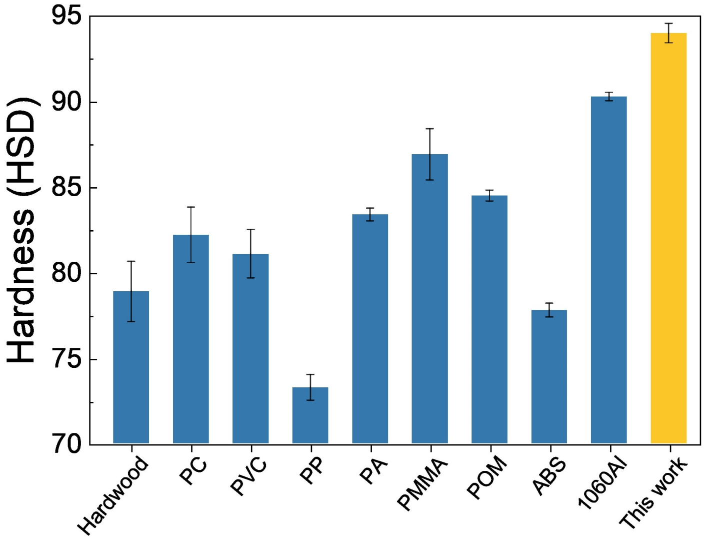


**Supplementary Figure 9 | Comparison of hardness.** Comparison of Shore D hardness number of the all-natural bioinspired structural material with other widely used materials. Error bars represent standard deviation.

**Supplementary Table 1 | Comparison of mechanical and thermal properties of all-natural bioinspired structural material with polyamide (PA)[1].**

| Properties | This work | PA |
|---|---|---|
| Ultimate strength (MPa) | 281 | 90-165 |
| Modulus (GPa) | 20 | 2.62-3.2 |
| Fracture toughness ($K_{Ic}$, MPa $m^{1/2}$) | 6.7 | 2.22-5.62 |
| Hardness (HSD) | ~94 | ~83 |
| Thermal expansion coefficient ($10^{-6}$ $K^{-1}$) | ~7 | ~147 |
| Thermal conductivity (W $m^{-1}$ $K^{-1}$) | ~0.53 | ~0.24 |

## Supplementary References


1. M. Ashby, *Materials Selection in Mechanical Design*. (Elsevier, Oxford, 2011).

2. Guan Q. F., et al. Lightweight, tough, and sustainable cellulose nanofiber-derived bulk structural materials with low thermal expansion coefficient. *Sci. Adv.* **6**, eaaz1114 (2020).
