# Supplementary material for: An all-natural bioinspired structural material for plastic replacement
Source: Nat Commun. 2020 Nov 3;11:5401. doi: 10.1038/s41467-020-19174-1 (PMC7642342; doi:10.1038/s41467-020-19174-1)
Supplement: Supplementary file 2 — Description of Additional Supplementary Files [file 41467_2020_19174_MOESM2_ESM.pdf]

Description of Additional Supplementary Files

File Name: Supplementary Data 1
Description: Three-dimensional reconstruction of the bioinspired structural material.
